# Molecular and Cellular Mediators of the Gut-Liver Axis in the Progression of Liver Diseases

**DOI:** 10.3389/fmed.2021.725390

**Published:** 2021-09-28

**Authors:** Alix Bruneau, Jana Hundertmark, Adrien Guillot, Frank Tacke

**Affiliations:** Department of Hepatology & Gastroenterology, Charité Universitätsmedizin Berlin, Campus Virchow-Klinikum (CVK) and Campus Charité Mitte (CCM), Berlin, Germany

**Keywords:** microbiota, liver diseases, immune cells, gut-liver axis, TLRs (Toll-like receptors), NAFLD (non-alcoholic fatty liver disease), NASH, PSC

## Abstract

The gut-liver axis covers the bidirectional communication between the gut and the liver, and thus includes signals from liver-to-gut (e.g., bile acids, immunoglobulins) and from gut-to-liver (e.g., nutrients, microbiota-derived products, and recirculating bile acids). In a healthy individual, liver homeostasis is tightly controlled by the mostly tolerogenic liver resident macrophages, the Kupffer cells, capturing the gut-derived antigens from the blood circulation. However, disturbances of the gut-liver axis have been associated to the progression of varying chronic liver diseases, such as non-alcoholic fatty liver disease, non-alcoholic steatohepatitis, and primary sclerosing cholangitis. Notably, changes of the gut microbiome, or intestinal dysbiosis, combined with increased intestinal permeability, leads to the translocation of gut-derived bacteria or their metabolites into the portal vein. In the context of concomitant or subsequent liver inflammation, the liver is then infiltrated by responsive immune cells (e.g., monocytes, neutrophils, lymphoid, or dendritic cells), and microbiota-derived products may provoke or exacerbate innate immune responses, hence perpetuating liver inflammation and fibrosis, and potentiating the risks of developing cirrhosis. Similarly, food derived antigens, bile acids, danger-, and pathogen-associated molecular patterns are able to reshape the liver immune microenvironment. Immune cell intracellular signaling components, such as inflammasome activation, toll-like receptor or nucleotide-binding oligomerization domain-like receptors signaling, are potent targets of interest for the modulation of the immune response. This review describes the current understanding of the cellular landscape and molecular pathways involved in the gut-liver axis and implicated in chronic liver disease progression. We also provide an overview of innovative therapeutic approaches and current clinical trials aiming at targeting the gut-liver axis for the treatment of patients with chronic liver and/or intestinal diseases.

## Introduction

The liver is a highly vascularized organ that receives ~75% of its blood supply from the enterohepatic circulation, delivering nutrients from the intestines, together with recirculating bile acids and gut microbiota-derived products. In turn, the liver provides signals to the gut by secreting bile, antimicrobial molecules in the bile ducts. The liver vasculature is made of fenestrated capillaries formed by liver sinusoidal cells, with intense biomolecule exchange between the blood compartment and the hepatic parenchymal cells. Liver resident macrophages, termed Kupffer cells (KCs), reside in these capillaries and play the roles of sentinels, sensing their microenvironment and catching cellular residues and microorganisms, thus maintaining homeostasis and an immunotolerant environment (1, 2). The intestinal mucosal and vascular barriers contribute to the communication between the gut and the liver, because they prevent microbiota and their metabolites from excessively spreading through the portal circulation in healthy conditions. The gastrointestinal tract shelters an ensemble of microorganisms including bacteria, fungi, archaea, viruses, and their genomes, all regrouped under the term of microbiome (3). Microbiota is a fundamental part of the gut, playing an essential role in digestion and bile metabolism, but also able to release a wide number of metabolites, peptides, and hormones capable of activating immune cells, thus continually shaping host immunity and metabolism (3, 4). The integrity of the gastrointestinal mucosa is then crucial to protect liver cells from exposure to gut-derived pathogen-associated molecular pattern molecules (PAMPs, e.g., bacteria and bacterial products), fatty acids and carbohydrates or modified bile composition (5).

The gut-liver axis is therefore an anatomical and functional connection existing through blood and bile circulation, integrating signals generated from environmental factors, diet, or microbiota (6). A healthy microbiota exerts protective effects (7, 8), highlighting the importance of considering therapeutic interventions targeting patients microbiota to slow down the progression of chronic liver diseases. However, growing evidence from clinical studies and experimental models show the involvement of gut-derived signals in the modulation of numerous liver diseases, including non-alcoholic fatty liver disease (NAFLD), non-alcoholic steatohepatitis (NASH), alcohol-associated hepatitis, cholestatic liver diseases, and in the progression to cirrhosis and hepatocellular carcinoma (HCC) (5, 6, 9, 10).

In a healthy liver, PAMPs are usually not harmful, since they are eliminated by KCs (1). However, in the context of acute or chronic liver inflammation, liver cell injury may induce cell death, releasing pro-inflammatory cytokines and chemoattractants, damage-associated molecular patterns (DAMPs), thus fueling chronic inflammation and innate immune cell recruitment (1). Furthermore, increased intestinal permeability associated with dysbiosis could result in bacteria translocation and higher presence of PAMPs or toxic bile acids, and increased fatty acid concentrations within the liver. Recognition of environmental immune signals, such as PAMPs, DAMPs, and gut-derived microorganisms by pattern-recognition receptors (PRRs) contributes to shaping myeloid immune cell phenotypes, thus participating in the progression of liver diseases (11). Hence, gut-derived signals can be aggravating factors of an innate immune response, most notably characterized by a potent infiltration of neutrophils and monocyte-derived macrophages, key in the orchestration of inflammatory response in acute and chronic liver diseases (6).

**Figure 1:**
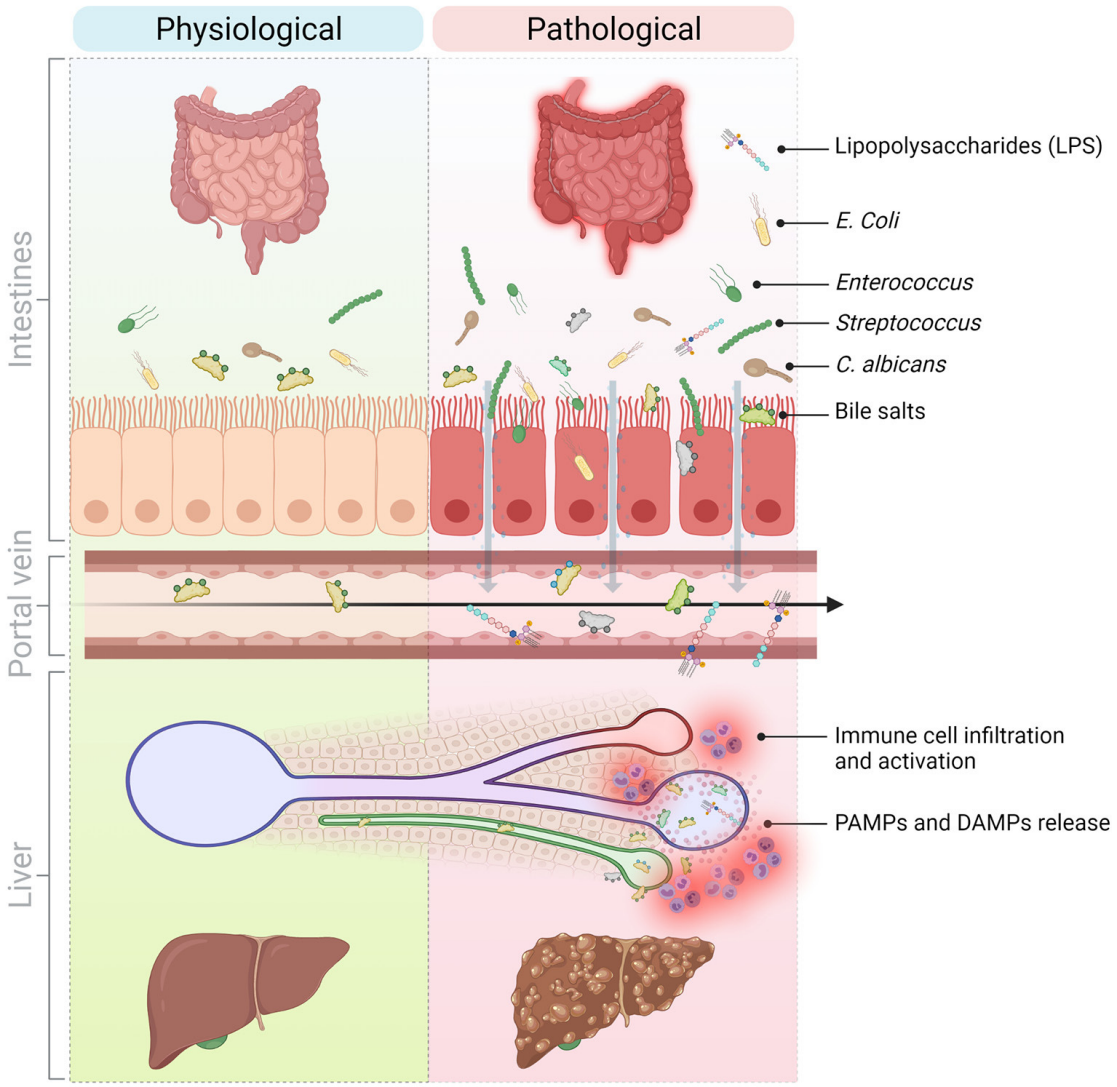
Molecular and cellular mediators of the gut-liver axis implicated in the progression of liver inflammation in chronic liver diseases. The communication between the liver and the gut is bidirectional. The liver secretes primary bile acids and antimicrobial peptides in the bile ducts while the gut contains host-, food-, and microbiota-derived antigens and metabolites. In normal conditions, these signals contribute to maintain physiological immune cell populations in the gut and are well-tolerated by the liver. However, in pathological conditions and because of a perturbed intestinal barrier (e.g., in NASH, NAFLD, PSC), DAMPs and PAMPs originating from the intestines translocate to the liver via the hepatic portal vein and thus, promote liver injury and inflammation and sustain liver disease progression. DAMPs, Danger-associated molecular patterns; LPS, Lipopolysaccharide; NAFLD, Non-alcoholic fatty liver disease; NASH, Non-alcoholic steatohepatitis; PAMPs, Pathogen-associated molecular patterns; PSC, Primary sclerosing cholangitis. Created with Biorender.

The liver also affects gut homeostasis. Indeed, bile acids and immunoglobulin A (IgA) secreted by the liver act as regulators of the gut microbiota (12) and immunity (13), for instance by preventing the colonization of pathogenic species and reshaping immune cell phenotypes in the gut. Thus, upon liver injury, a modified bile acid pool might in turn affect microbiota composition and gastrointestinal inflammation (Figure 1).

For all these reasons, deciphering the molecular mechanisms involved in immune cell recruitment and activation in the liver and the intestine is of great interest in developing promising therapeutic approaches. In this review, we summarize the current knowledge about molecular and cellular mediators of the gut-liver axis and their involvement in the progression of liver diseases. We also detail the most recent therapeutic options and perspectives to treat patients suffering from various liver diseases, by focusing on targeting actors of the gut-liver axis.

## Involvement of the Gut-Microbiota in Liver Diseases

Our understanding of the composition and functions of the gut microbiota in physiological conditions is in constant evolution. Several national or multinational studies contributed to the identification of three main enterotypes (14) present through all populations and continents, *Bacteroides, Prevotella, Ruminococcus*, comprising the main bacterial phyla: *Firmicutes, Bacteroidetes, Actinobacteria, Proteobacteria*, and *Verrucomicrobia* (15). The gut microbiome is highly variable among individuals and depends on many elements, including age, birth mode, diet, geography, exercise, and other lifestyle factors, such as alcohol consumption and exposure to antibiotics (3, 16). Over the last decade, many research articles highlighted the role of dysbiosis in liver diseases (Table 1). As discussed below, disruption in gut microbiota homeostasis can affect bile acid metabolism, intestinal permeability, short chain fatty acids (SCFAs) availability and consequently alter alcohol, glucose and lipid metabolism, dietary energy utilization, along with promoting liver injury, and inflammation. However, whether intestinal dysbiosis is part of the causes or a result of liver diseases remains an unanswered question in many cases.

**Table 1:** Summary of studies analyzing microbiota in chronic liver diseases.

**Disease**	**Characteristic changes in the gut microbiota (dysbiosis)**	**Sample**	**References**
PBC	↓ *Sutterella, Oscillospira* and *Faecalibacterium* and ↑ *Haemophilus, Veillonella, Clostridium, Lactobacillus, Streptococcus, Pseudomonas, Klebsiella*	Stool	(17)
PSC	↑ *Veilloneilla* ↑ *Escherichia, Lachnospiraceae, Megasphera* and ↓ *Clostridiales II* ↓ *Clostridiales II* ↑ Ruminococcus and Fusobacterium, ↓ Dorea, Veillonella, Lachnospira, Blautia, and Roseburia ↑ *Enterococcus, Streptococcus, Lactobacillus* ↑ *Rothia, Enterococcus, Streptococcus, Veillonella*, ↑ *Trichocladium griseum, Candida spp*. ↓ *Firmicutes spp*. (*Faecalibaterium* and *Ruminococcus*) except ↑ of *Veilloneilla*, ↑ Proteobacteria; ↓ *S. cerevisiae* and ↑ *Exophiala*	Stool Colon biopsies Colon + Ileum Stool Stool Stool Stool Stool	(18) (19) (20) (21) (22) (23) (24) (25)
ALD	↑ *Candida*, ↓ *Epicoccum*, ↓ *Galactomyces* ↑ *Bifidobacterium, Streptococcus spp, Lactobacillus spp* ↓ Prevotella, Paraprevotella, and Alistipes	Stool Stool	(26) (27)
NAFLD	↑ Escherichia coli and Bacteriodes vulgatus, ↓ *Ruminococcus spp., Eubacterium rectale, Faecalibacterium prausnitzii* ↓ virus and bacteriophage diversity, ↑ *Escherichia, Enterobacteria*, and *Lactobacillus* phage ↑ *Gemmiger*, ↓ Faecalibacterium, Bacteroides and Prevotella ↑ *Gemmiger*, ↓ *Bacteroides*	Stool Stool Stool Stool	(28) (29) (30) (31)
NASH	↑ *Bacteroides*, ↓ *Prevotella* ↓ *Firmicutes* and *Clostridiales* (*Faecalibacterium* and *Anaerosporobacte)*, ↑ *Bacteroidetes* (*Parabacteroides* and *Allisonella)* ↑ Proteobacteria, *Bacteroidetes*, ↓ *Actinobacteria, Firmicutes*	Stool Stool Stool	(32) (33) (34)
Cirrhosis	↑ *Veillonella spp*. and *Streptococcus spp*., ↓ *Bacteroidetes* and *Firmicutes* ↓ *Lachnospiraceae, Ruminococcaceae* and *Blautia* ↑ *Staphylococcaeae, Enterobacteriaceae, Enterococcaceae* and *Enterococcaceae*, ↓ *Lachnospiraceae, Ruminococcaceae* and *Clostridiales XIV* ↑ *Candida spp*.	Stool Stool Stool Duodenal fluid	(9) (35) (36) (37)
HCC	↑ *Bacteroide*, ↓ *Bifidobacterium, Blautia* ↑ *Escherichia coli* Presence of *H. pylori*	Stool Stool Liver	(38) (39) (40)

*ALD, Alcohol-related liver disease; HCC, Hepatocellular carcinoma; NAFLD, Non-alcoholic fatty liver disease; NASH, Non-alcoholic steatohepatitis; PBC, Primary biliary cholangitis; PSC, Primary sclerosing cholangitis; Underlined: fungi. ↓ decrease ↑ increase*.

### Cholangiopathies

Cholestatic diseases or cholangiopathies encompass several conditions, from pediatric genetic liver diseases, e.g., progressive familial intrahepatic cholestasis (PFIC), to adult idiopathic or genetic diseases including primary biliary cholangitis (PBC), and primary sclerosing cholangitis (PSC).

Notable changes in the composition of the gut microbiota in patients with PSC and PBC have been reported, suggesting a role of microbiota in their pathogenesis (41). Enriched amounts of some species, including *Veillonella, Streptococcus*, and *Enterococcus* or depletion in *Clostridiales II* have been described in feces or mucosal biopsies of patients in several cohorts (18–20, 22). Moreover, the strong association of Inflammatory Bowel Disease (IBD) and PSC is well-known, in particular in the Northern European population with ~80% of PSC patients suffering from IBD (42). It is plausible that dysbiosis and liver inflammation are functionally linked, as proposed by the leaky gut hypothesis. Indeed, supporting this hypothesis, a recent study showed an increased recruitment of CD11b^+^CD11c^−^Ly6C^+^ macrophages in the liver, associated with bacteria homing after induction of colitis in PSC mouse models (43). However, the gut-liver axis is bidirectional, and thus an impaired bile acid flux or modified bile acid composition could very well alter the microbiota in return (e.g., by promoting the colonization of invasive bacterial populations) and may thereby provoke a much more harmful translocation of PAMPs to the liver, thus aggravating liver injury in a negative feedback loop. The *Mdr2*^−/−^ mouse model is commonly used as a model for PSC. As these mice lack phospholipids in its bile, toxic free bile acids and cholesterol crystals will trigger cholangiocyte injury. Tedesco et al. demonstrated that these mice spontaneously display an increased intestinal permeability and dysbiosis with an enrichment in *Lactobacillus sp*. associated with increased IL-17 in the serum (44).

Recently, different studies demonstrated that besides bacteria dysbiosis, PSC patients also suffer from fungi dysbiosis. According to a French study by Lemoinne et al., PSC patients with associated IBD display a specific signature different from PSC patients without IBD or patients with IBD only (25). On the contrary, in a German cohort, no differences were found between PSC patients suffering colitis or not. However, they confirmed Lemoinne et al. data concerning an increase of *Candida* species and of the fungal class *Sordariomycetes* for all PSC patients compared to the healthy group (24).

### Non-alcoholic Fatty Liver Disease and Non-alcoholic Steatohepatitis

NAFLD is the most frequent cause of chronic liver disease worldwide. NAFLD is commonly associated to metabolic syndrome, insulin resistance, type 2 diabetes, and obesity. The term NAFLD refers to a wide spectrum of conditions, from simple liver steatosis to NASH. NASH is characterized by chronic inflammation, fibrosis, hepatocellular injury, and can progress to cirrhosis and HCC (45).

There is evidence for the involvement of several components of the gut-liver axis, e.g., microbiota dysbiosis, modification in the gut barrier permeability, bile acid metabolism changes, and SCFAs in the progression of the NAFLD and NASH (46).

The first report suggesting an impact of gut microbiota in human NAFLD dates back to the 80s (47). Consequently, other studies explored the roles of gut microbiome in patients with NASH. Small intestinal bacterial overgrowth (SIBO) in NASH groups compared to controls has been reported by Wigg et al. (48) as well as Shanab et al. (49), while several studies characterized dysbiosis in more detail. An increase in *Bacteroidetes* phylum, colonization by pro-inflammatory *Proteobacteria, Enterobacteriaceae*, and *Escherichia* and decrease in *Firmicutes* (including *Prevotella, Faecalibacterium* species) are the most common changes observed in NAFLD and NASH patients (32–34). Boursier et al. were able to link fecal microbiota alterations with the severity of NAFLD lesions, based on changes in *Bacteroides* and *Ruminococcus* abundance (32). Serum and hepatic bile acid concentrations can be modified in NASH patients, and the latter could have an effect on the progression of fibrosis (50).

An increase of the gut barrier permeability as a result of dysbiosis leads to higher bacterial translocation and elevated levels of LPS reaching the liver, leading to PRR activation and immune cell recruitment, thus sustaining liver inflammation.

### Alcohol-Related Liver Diseases

Excessive alcohol drinking is a major cause of liver damage and deaths worldwide (51). Involvement of gut dysbiosis in the severity of liver injury in alcohol-related liver diseases has long been emphasized in both patients and animal models (52). Following 6 weeks of alcohol feeding, mice have a loss of bacterial diversity characterized by a shift in phyla with more *Proteobacteria, but* less *Bacteroidetes, Firmicutes*, and *Lactobacillus* (53). Accordingly, in a mouse model, 3 weeks of alcohol exposure were sufficient to observe an increase in plasma LPS. In the same study, the authors observed bacteria overgrowth, which they attributed to a downregulation of mouse antimicrobial proteins Reg3b and Reg3g (54). Ethanol-induced gut microbiota alterations were also associated with changes in metabolic profiles, including an increase in intestinal levels of SCFAs. This mouse data has been confirmed in patient cohorts, showing that alcohol is one of the main factors contributing to modifications in the gut microbiota. For instance, an enrichment in pro-inflammatory *Enterobacteria* such as *Escherichia* and *Klebsiella* and a decreased abundance in butyrate-producing species which have an anti-inflammatory protective effect were reported (27, 55). 16S sequencing from patients with alcohol-associated hepatitis highlighted a drastic increase of *Enterococcus faecalis* in stool samples compared with controls. Duan et al. identified cytolysin, a bacterial toxin secreted by *E. faecalis*, in stool samples of these patients and showed that its presence is associated with a worse clinical outcome and a higher death rate. Moreover, they demonstrated that in a mouse model infected with a cytolitic *E. faecalis* strain followed by an ethanol diet *E. faecalis* can be detected in the liver and is associated with increased liver inflammation. *In vitro*, the cytolitic *E. faecalis* promotes primary hepatocytes cell death, offering a possible explanation for the ethanol-liver injury induced by this bacteria strain (56).

Fungi also play a role in the development of alcohol-related liver disease. Indeed, Yang et al. showed that chronic ethanol administration is responsible for a fungal dysbiosis and elevated plasma levels of β-glucan in mice (26). They presented evidence for similar modifications in the composition of fecal mycobiome for patients with chronic alcohol abuse. The diversity and richness of fungal species is reduced in alcohol-dependent patients compared to healthy controls. Additionally, an overgrowth of *Candida* species, mainly *Candida albicans* is observed (26).

### Cirrhosis

Cirrhosis is the end-stage of chronic liver diseases and is associated with dysbiosis and a disruption of the intestinal barrier, partly due to portal hypertension. Portal hypertension does not only promote neo-angiogenesis and intestinal permeability, but also increases intercellular spaces between enterocytes and affects microvilli density in patients with cirrhosis (57), thus allowing PAMPs to easily reach the liver and accelerate the pre-existing hepatic inflammation.

The gut microbiome of a Chinese cohort of 98 patients has been sequenced from stool samples and the authors found out that species decreasing the most belong to *Bacteroidetes* and *Firmicutes* phyla, while *Streptococcus* and *Veillonella spp*. have the greatest increase (9). They propose a patient discrimination index (PDI) relying on 15 gene markers, all related to gut microbiota, which could be used to diagnose liver cirrhosis in a non-invasive way. Bajaj et al. obtained similar results in patients with varying cirrhosis severity: an enrichment in pathogenic taxa, *Staphylococcaceae, Enterobacteriaceae*, and *Enterococcaceae* along with a decrease in *Lachnospiraceae, Ruminococcaceae*, and *Clostridiales XIV* (35, 36). By calculating a cirrhosis dysbiosis ratio (CDR), the same authors correlated the severity of cirrhosis with the changes in patient's microbiota. The reduced species are important for the production of SCFAs, thus reducing the intestinal inflammation and protecting the mucosa. A lower amount of SCFAs might also explain the disruption of the intestinal barrier as well as the release and translocation of PAMPs (including LPS) to the liver, and thereby contribute contribute to the disease severity and occurrence of complications in patients (36). Moreover, this dysbiosis is responsible for modifications in the bile acid pool in the gut, caused by a depletion in certain bacteria species involved in the regulation of primary bile acids conversion (35).

A clear correlation exists between fungal infection, higher inflammation, and increased mortality rate in patients with end stage liver disease (58). Several *Candida* species were detected with 18S rRNA gene-based PCR method in a cohort of patients with cirrhosis (37). A more recent study linked fungal infections in cirrhotic patients with a weakened ability of their neutrophils to kill *C. albicans* (59). Mechanisms involved in the progression of cirrhosis or complications in patients following fungal dysbiosis remain mostly unexplored but are of interest to develop innovative and efficient therapies.

### Hepatocellular Carcinoma

Hepatocellular carcinoma (HCC) represents about 80% of primary liver cancer cases worldwide (60). Increased intestinal permeability and significant modifications in the microbiota profile of HCC patients strongly advocate for a role of the gut-liver axis in the progression of HCC (38). Along with a disturbed intestinal mucosa, LPS was shown to be increased in HCC patient serum, and there has been evidence of bacterial translocation, which correlate with chronic inflammation characterized by more CD14^+^PD-L1^+^ circulating monocytes and a specific cytokine and chemokine signature in the HCC group. The deficit in anti-inflammatory species *Bifidobacterium* or *Blautia* could explain this enhanced intestinal and hepatic inflammation (38). Depending on the studies, the dysbiosis is characterized by increased *Escherichia coli* (39), increased *Bacteroides* (38), and *H. pylori* presence has even been detected in liver samples from HCC patients (40). Rao et al. suggested using patient's microbiota signature from tongue swab as a non-invasive tool for HCC diagnosis (10).

Several studies using animal models relate to this clinical data: in a diethylnitrosamine (DEN) model of rat HCC, authors noticed a decrease in *Bifidobacterium, Enterococcus*, and *Lactobacillus* species in the gut (61). Moreover, colonization of the gut with *Helicobacter hepaticus* in aflatoxin B1 (AFB1)-induced liver cancer mouse model is linked with a poor prognosis and the severity of inflammation, thus promoting carcinogenesis (62).

## Gut-Derived Metabolites and Molecular Pathways

Microbial metabolites and bile acids shape immune cell maturation and homeostasis and contribute to maintain intestinal barrier integrity. Thus, modifications in the microbiota or their metabolite profiles can alter immune response and trigger inflammation in the gut and in the liver. Furthermore, modifications in bile acids, SCFAs or tryptophan metabolites have been described in the pathogenesis of several chronic liver diseases.

### Molecular Mediators in the Gut-Liver Axis

#### Bile Acids

Bile acids have a key role in homeostasis as they contribute to the absorption of dietary fats and liposoluble vitamins and prevent commensal bacteria over-growth or colonization of the intestines by pathogenic bacteria species (63). Bile acids are secreted by hepatocytes through the ATP-dependent transporter called ABCB11, and are derived from two different origins; primary bile acids (5% of total bile acids) are *de novo* synthesized from cholesterol and secondary bile acids (95%), which are deconjugated by gut microbiota and recycled daily after ileal reabsorption through the entero-hepatic circulation (12). In homeostatic situations, the ratio between glyco-conjugated and tauro-conjugated bile acids synthesized in the liver is tightly regulated. Similarly, the deconjugation of bile acids in the colon by anaerobic bacteria is under strict control since secondary bile acids are more hydrophobic and thus more toxic for the intestinal and hepatic epithelial cells (63, 64). Thus, (1) a modification in the ratio of synthesized bile acids could change the antimicrobial properties of bile and alter the gut microbiota as well as having a deleterious effect on hepatocytes and cholangiocytes membrane, generating apoptosis, and inflammation in the liver; and (2) an intestinal dysbiosis might be responsible for the increased generation of toxic bile acids by bacteria (12).

Modifications of the bile acid profile in the plasma of PSC patients have been evidenced and are clinically associated with hepatic decompensation (65). Torres et al. were able to demonstrate a relationship between stool bile acids profile and microbiota composition in patients with PSC associated to IBD compared to patients suffering from IBD alone, thus suggesting an effect of bile acids on the microbiota composition of PSC patients (21). Similar findings have been made with circulating bile acids from NAFLD and NASH patients (66, 67). Moreover, in a mouse model of high fat-diet (HFD), the presence of hydrophobic bile acids is correlated with liver inflammation and bacteria dysbiosis, and promotes carcinogenesis (68). Along with dysbiosis and stronger colon inflammation, Xie et al. have reported increased expression of several genes involved in bile synthesis in a model of alcohol-related liver disease (68), while others indicate higher concentrations of total fecal bile acids as well as modifications in the primary to secondary bile acids ratio in patients actively drinking (69).

Bile acids are recognized by several receptors in a wide variety of cells and can regulate bile acid synthesis and immune cell activation. Bile acids can directly bind the Farnesoid X Receptor (FXR) which will then be translocated to the nuclei and inhibit bile acids synthesis. FXR is expressed in hepatocytes, enterocytes, and regulates lipid metabolism, glucose metabolism, and inflammation on top of bile acids synthesis (70). Targeted inhibition of intestinal FXR helped reducing hepatic lipid droplets in a HFD model, thus protecting mice from hepatic steatosis. Moreover, authors showed an overexpression of FXR and its downstream effectors in the intestine of obese humans compared with lean controls, thus suggesting a role of FXR in the pathogenesis of metabolic syndromes in patients (71). Indeed, modified bile acid composition and elevated plasma bile acids levels in NAFLD patients negatively influence FXR signaling, which impacts bile acid synthesis, lipid and glucose metabolism, and inflammation, thus potentially contributing to hepatic injury. Additionally, expression of downstream effectors of intestinal FXR is decreased in NAFLD and NASH patient biopsies (72). In rodents fed with HFD, intestinal FXR is downregulated and the use of an FXR agonist, obeticholic acid (OCA), helped preserve the gut barrier integrity, thus decreasing PAMPs amount in the liver (73). Decreased FXR expression also correlates with fibrosis and NAFLD activity score (74). Moreover, Pathak et al. showed that intestinal FXR activation is responsible for microbiota composition modifications, which will in return activate TGR5 signaling, improving glucose and lipid metabolism in an obesity mouse model, thus suggesting a role in NAFLD and NASH pathogenesis (75).

**Figure 2:**
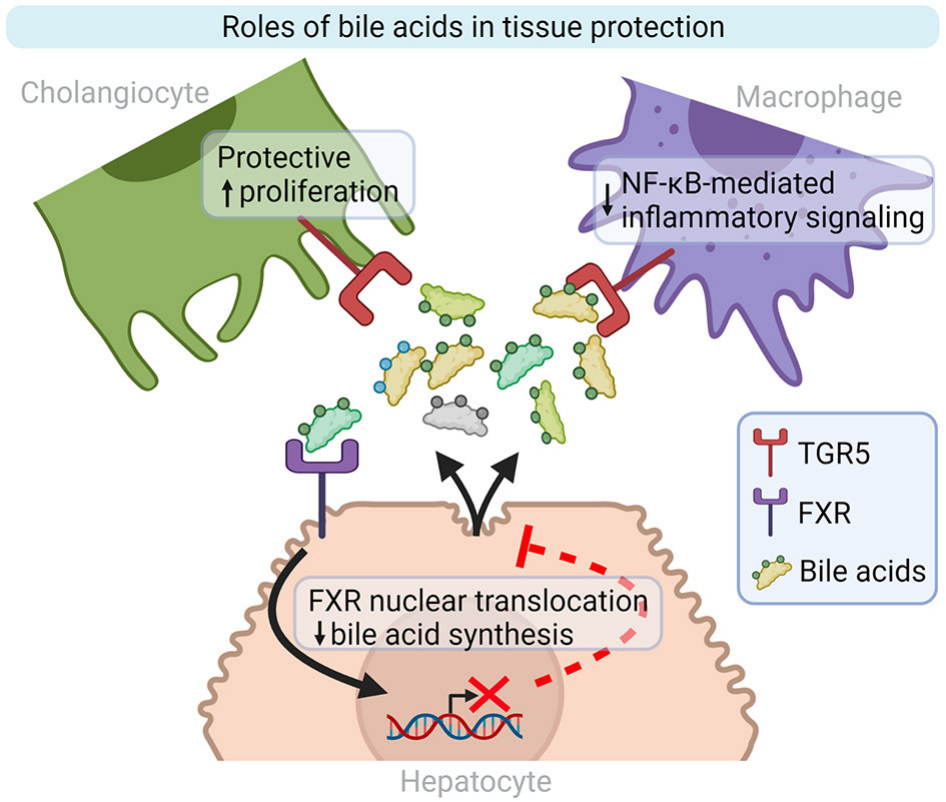
Bile acid roles in inflammation and tissue repair modulation. TGR5 and FXR are two well-characterized bile acid receptors implicated in liver homeostasis. TGR5 activation on cholangiocytes and macrophages stimulates cell proliferation and decreases inflammatory signaling, respectively. Bile acid synthesis by hepatocytes is repressed by FXR activation. FXR, Farnesoid X-Activated Receptor; NF-κB, Nuclear factor kappa-light-chain-enhancer of activated B cells; TGR5, also known as GPBAR1, G-protein-coupled bile acid receptor. Created with Biorender.

TGR5 is a plasma membrane associated protein expressed by cholangiocytes, immune cells -including KCs- and hepatic stellate cells (HSC), mostly activated by hydrophobic bile acids. In reaction to LPS, TGR5 represses the secretion of pro-inflammatory cytokines by macrophages through an NF-κB dependent pathway (Figure 2) (70). Leonhardt et al. showed that patients with liver failure display a specific serum bile acids profile responsible for TGR5 activation in monocytes and correlated with increased mortality. Indeed, monocytes from healthy controls treated with TGR5-activating bile acids that were then stimulated with LPS, exhibit a drastically diminished pro-inflammatory phenotype (76). In a *Tgr5*^−/−^ mouse model fed with alcohol, Spatz et al. observed increased liver injury, inflammation and steatosis compared to WT mice fed with alcohol. Those mice also display increased dysbiosis, independent from the alcohol uptake, massive macrophage infiltration (77). Moreover, TGR5 is downregulated in PSC patients and *Mdr2*^−/−^ mice cholangiocytes, which promotes biliary injury and liver inflammation. Norursodeoxycholic acid (norUDCA) treatment can increase TGR5 expression, and thus attenuate biliary inflammation phenotype in *Mdr2*^−/−^ mice (78). The treatment of HFD model with TGR5 agonists is able to reverse steatohepatitis in mouse and to generally improve liver histology (79, 80). In general, studies in animal models suggest that those receptors may be downregulated by ethanol consumption (81), liver inflammation, gut dysbiosis, or activation of NF-κB (82), and that the use of TGR5 and FXR agonists could be beneficial for patients (83–85).

#### Bacterial and Fungi Products

Innate immune cells can recognize a wide variety of PAMPs released by bacteria, viruses or fungi. Among them, LPS (from Gram-negative bacteria), bacterial flagellin, bacterial/viral nucleic acids (ssRNA, dsRNA, CpG DNA), peptidoglycans (Gram-positive bacteria) β-glucans from fungi can all be recognized by PRRs and involved in the progression of liver diseases (6). As stated above, elevated serum levels of LPS or LPS-binding protein have been described in nearly all liver diseases: Alcohol-related liver diseases (6), NAFLD and NASH (46, 86), HCC (38, 61), PSC (87), indicating an increase of intestinal permeability and suggesting that signals derived from a diseased liver (e.g., modified bile acids, pro-inflammatory cytokines) affect the intestinal barrier integrity.

Bacteria can also interact with the host via secretion of metabolites, either derived from bacteria metabolism like tryptophan, or host molecules modified by bacteria such as bile acids (see above). The gastrointestinal tract is a central place for tryptophan metabolism, where it can directly and indirectly be metabolized to indoles and their derivatives by microbiota. Indoles are able to decrease macrophage production of pro-inflammatory cytokines and inhibit macrophage migration (86). Discovery of a perturbed tryptophan metabolism in patients suffering from IBD or colitis suggests common mechanisms for patients suffering of liver diseases associated with intestinal dysbiosis (88). In mice that were subjected to a model of alcohol-related liver disease and transplanted with patient's microbiota, targeting the tryptophan pathway was effective in reducing liver injury (89). Moreover, in order to determine a microbial signature in HCC, patient's stool microbiota was sequenced. Albhaisi et al. found no differences in the overall microbial diversity for cirrhotic patients who would develop HCC. However, specific changes in species involved in tryptophan metabolism were detected (90). Even though, research for new biomarkers in patients or animal models highlight the involvement of tryptophan and its metabolites in the pathogenesis of PSC (91) and NASH (92), the causal link with microbiota dysbiosis yet remains to be demonstrated.

In the past years, the interest for the mycobiome has been rising. Despite being at lower abundance compared to bacteria, commensal fungi are essential for tissue homeostasis and regulate many physiological processes (93). Fungal dysbiosis has been observed in several liver diseases as NASH (93), PSC (24, 25), alcohol-related liver diseases and cirrhosis (37, 58). Fungal dysbiosis occurs following chronic ethanol administration in mouse and is responsible for elevated plasma levels of β-glucan, and hepatocyte damage. It could activate liver resident macrophages through chitin or β-glucan (26, 93).

#### Short-Chain Fatty Acids

SCFAs produced by gut microbiota, in particular by *Firmicutes*, are essential actors in the maintenance of gut homeostasis through strong anti-inflammatory and anti-proliferative effects (94). Several SCFAs are generated by bacteria from dietary fibers, the predominant ones are butyrate, acetate and propionate (94). In patients with ulcerative colitis as well as colitis mouse models, changes in SCFAs concentration (in particular butyrate) are responsible for the activation of several pro-inflammatory pathways, including activation of the inflammasome (NLRP3) pathway with increased IL-18 secretion, increased production of pro-inflammatory cytokines like IFN-γ, IL-5, IL-8, IL-10, and IL-13 but also downregulation of NF-κB signaling, all of which have been linked to gut microbiota changes (4, 95). Dysbiosis observed in patients with chronic liver disease is often associated with a loss of bacterial species producing butyrate, thus explaining a reduction in SCFAs in several patient cohorts (27, 96). There is growing evidence that T-cell immunity can be regulated by gut-microbiota through SCFAs. On one hand, SCFAs can induce IL-10 secretion by CD4^+^ Th1 cells, hence protecting mice from colitis (97), but on another hand, *in vitro*, SCFAs have been shown to affect the balance between Th17 and Treg cells by affecting peripheral blood mononuclear cells (PBMCs) production of cytokines involved in T cell differentiation (98).

Increase in SCFAs can also be deleterious. Indeed, in NAFLD-HCC patients, dysbiosis has been associated with an enrichment of five bacteria species, all linked to an elevated SCFAs production compared to control or NAFLD-cirrhosis groups. Behary et al. connected this specific bacteria profile of NAFLD-HCC patients, with elevated SCFAs concentration and T cell response, with an expansion of IL-10^+^ Treg cells and a reduction of cytotoxic CD8^+^ T cells in an *ex vivo* model (99). Involvement of SCFAs in the pathogenesis of cholestatic liver cancer has been highlighted by Singh et al. in a model of gut dysbiosis in mice fed with enriched amounts of SCFAs. They showed that feeding with increased SCFAs is responsible for an even stronger intestinal dysbiosis in these mice as well as increased cholestatic liver injury, leading to HCC after 6 months. After 2 weeks of feeding, the mice displayed elevated bile acids in the serum, and at 4 weeks, hepatocyte apoptosis was elevated and triggered a massive neutrophils infiltration (100). A recent study correlated the presence of SCFAs with fibrosis in PBC patients, however the molecular roles of SCFAs in the pathogenesis of PBC have not been studied to this day (101).

#### Micro-RNA (miRNA)

Perturbation of the gut-liver axis can be responsible for changes in the expression of miRNAs. miRNAs are small single-stranded, non-coding RNAs involved in the silencing of protein-coding messenger RNAs (mRNAs) through translational repression and mRNA degradation (102). Modifications of miRNAs expression is connected with several chronic liver diseases, including NAFLD (103, 104), alcohol-related liver disease (105), cirrhosis, and HCC (106). Moreover, the impact of diet or gut-microbiota derived metabolites in the regulation of miRNAs expression in the gut and in the liver was demonstrated by multiple studies (106–109).

miR-22 expression is known to be down-regulated in HCC patients (109). A loss of butyrate-producing bacteria has been described in several HCC cohorts and may explain the link between intestinal dysbiosis and hepatic miR-22 down-regulation in those patients (108, 109). *In vitro*, butyrate induces miR-22 expression in the Huh7 hepatoma cell line. Moreover, Pant et al. showed that following miR-22 activation by butyrate, a SCFA produced by bacteria, ROS production is increased, cells undergo apoptosis and cell proliferation is inhibited (109).

In a rat model of NASH, gut dysbiosis, and increased intestinal permeability aggravates liver inflammation, and the authors observed modifications in miRNAs expression. For example, miR-122 expression is increased in the serum of NASH and NAFLD patients, while its hepatic expression is decreased. On the contrary, miR-146b expression is reduced in NASH patient serum and increased in tissue. Those two miRNAs are known for acting on the hepatic stellate cells, promoting fibrosis (110, 111). In a HFD model, the authors observed that HFD induces a microbiota dysbiosis and a hepatic downregulation of miR-122 and upregulation of miR34a as compared to mice fed with standard diet (112). A correlation analysis in HFD induced NAFLD mouse model, showed that miR-34a was associated with alterations in the gut microbiota, in particular with modifications in Firmicutes (113). Santos et al. used a mouse model lacking miR-21 and showed that when challenged with bile duct ligation (BDL), the absence of miR-21 protects those mice from liver injury and fibrosis (114). Those results are similar to those from Blasco-Baque et al., who used the same miR-21 KO mouse and demonstrated that they are protected from NASH with significant reduction of steatosis, fibrosis and inflammation (115). In both cases, the authors were able to link the hepatic expression of miR-21 and other miRNAs with gut microbiota changes. Indeed, LPS regulates the expression of miR-21, miR-181a, and miR-666 in a dose-dependent manner (115). Moreover, miR-21 deletion is responsible for gut microbiota modifications, which seem to have a protective effect in mice and to prevent a detrimental macrophage pro-inflammatory phenotype in the gut (114). A direct relationship also exists between LPS concentration and miR-146a-5p expression in the progression of liver fibrosis, indicating once again an indirect link between miRNAs and gut microbiota (116). Several miRNAs induce macrophage activation and contribute to inflammation and progression in liver diseases (117).

Also, in female mice, dysbiosis is not as severe as in male mice, leading to less toxic modification in the bile acids profile, which is likely due to protective role of estrogens (118). Furthermore, female mice have higher levels of the bile acid receptor FXR compared to male, which seems to increase the expression or miR-26a and miR-122, known to have tumor-suppressive effects in a murine NASH model (118). miRNAs are also known to be regulators of TLRs in immune cells (119), a family of receptors involved in the recognition of PAMPs as detailed in this review.

### Recognition and Processing of Gut-Derived Signal in Hepatic and Immune Cells

#### Pattern Recognition Receptors and Signaling Pathways

**Figure 3:**
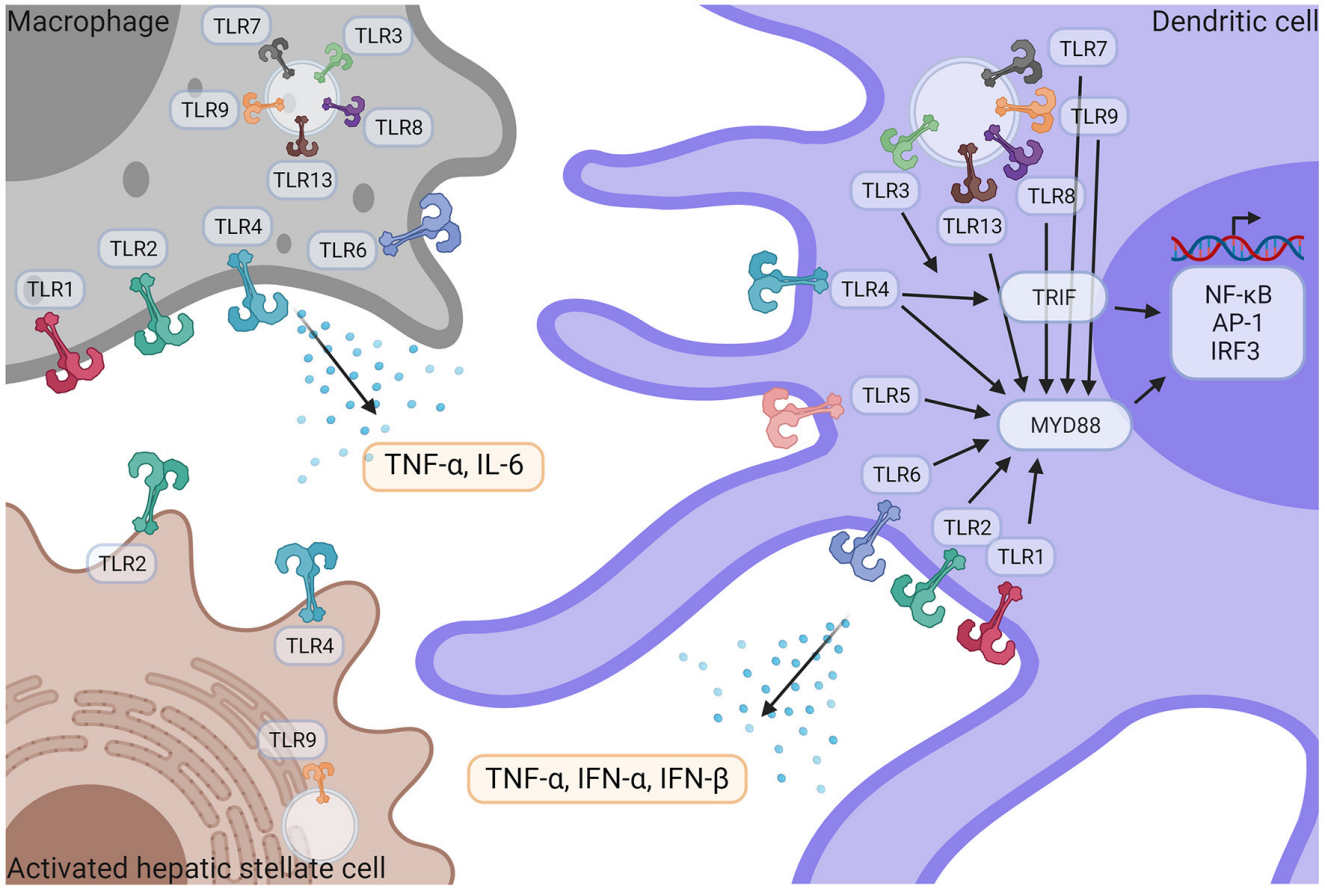
TLR signaling in liver immune pathogenesis. Macrophages and DCs express TLR1,−2−3−4−6−7−8−9−13 and TLR5 is only expressed in DCs. HSCs express TLR2, TLR4, and TLR9. Following activation by their respective ligands, TLRs induce TRIF and/or MyD88 signaling pathways, leading to the activation of NF-κB, AP-1, and IRF3 transcriptional factors, followed by the release of pro-inflammatory cytokines such as TNF-α, IFN- α, IFN-β and IL-6. IFN-α, Interferon α; IFN-β, Interferon β; IL-6, Interleukin 6; NF-κB, nuclear factor kappa-light-chain-enhancer of activated B cells; TNF-α, Tumor necrosis factor α; TLR, Toll like receptor. Created with Biorender.

With 10 members identified in humans, Toll-like receptors constitute the main family of PRRs. They have a fundamental role in the recognition of pathogens and pathogen-derived motifs (i.e., PAMPs). TLRs are expressed by hepatic stellate cells, liver parenchymal cells such as hepatocytes, cholangiocytes, as well as a wide variety of immune cells, including resident and circulating macrophages, dendritic cells (DCs) and neutrophils (Figure 3) (5). TLR signaling pathways are involved in maintaining homeostasis, and dysregulations of those pathways are involved in aberrant inflammatory reactions and autoimmune diseases. TLR1 (binding bacterial lipoproteins), TLR2 (bacterial and fungi lipoproteins), TLR4 (LPS), TLR5 (flagellin), and TLR6 (bacterial lipoproteins), are plasma membrane localized TLRs while TLR3 (dsRNA), TLR7, and TLR8 (ssRNA) TLR9 (unmethylated CpG containing ssDNA) and TLR13 (bacterial ribosomal DNA) are found in endosomes. Despite different cellular localizations, they share common signal transduction pathways. Upon TLR activation, two main molecular pathways can be induced: one mediated by TRIF (TLR3, TLR4) and another one involving Myd88 (all TLRs but TLR3) (120). They will both lead to the activation of transcriptional factors NF-κB, AP-1, and IRF3 thus promoting the expression and release of several pro-inflammatory cytokines: TNF-α, IL-1β, IL-6 as well as IFNs. TRIF can also promote RIPK3-dependent necroptosis (120). Isolated KCs strongly express all TLRs except TLR5, and KCs respond to all TLR ligands mainly by secreting TNF-α and IL-6 (121). Activated HSCs express TLR2, 4, and 9. Murine hepatic DCs express all TLRs at various levels and IFN-α, IFN-β and TNF-α are released after activation with TLR3 agonists, TLR3,−4,−7,−8, and−9 and all TLR agonists (121).

#### Cholangiopathies

Several groups demonstrated that mouse and human cholangiocytes express TLR2, 4, and 5, allowing them to recognize the PAMPs present in bile. Upon stimulation with bacterial components, cholangiocytes are able to trigger NF-κB pathway activation and release of IL-6 *in vitro* (122). The role of TLRs has been demonstrated in cholangiocytes for PSC and PBC patients, but little is known concerning their activation in immune cells and their role in the pathogenesis of these cholangiopathies (5).

#### Alcohol-Related Liver Disease

It is well-known that serum LPS levels are elevated in alcohol-related liver disease patients and are correlated with liver injury (52).

Expression levels of TLR2 and TLR4 were assessed in peripheral blood monocytes of patients suffering from alcohol-related liver disease. Even though no significant difference was detected between the groups, the authors described a diminution of TLR2-mediated immune response in the alcohol-related liver disease group compared to controls following stimulation of the cells with bacterial products (123). In another study, overexpression of TLR2, 4, and 9 has been associated with neutrophil dysfunction for patients suffering from alcohol-associated hepatitis (124).

Mice fed with ethanol for 10 days display an overexpression of liver TLR1, 2, 4, 6, 7, 8, and 9 at the mRNA level, resulting in an upregulation of TNF-α (125). KCs from rats exposed to ethanol upregulate TLR2 and TLR4 compared to control groups (126).

Feeding of WT mice with ethanol and unsaturated fat has a negative impact on liver injury and steatosis compared to ethanol and saturated fat feeding. This combination clearly damages the intestinal barrier, thus increasing LPS levels in mouse serum, which aggravates hepatic inflammation, characterized by a massive macrophage infiltration but also upregulation of all TLRs in the liver. It is unfortunate that the cell types overexpressing TLRs have not been characterized in this study (127). In a recent study, depletion of *Roseburia spp*. was associated with alcohol consumption and modifications in fecal SCFAs in patients, while *Roseburia spp*. supplementation in ethanol-fed mouse group could weaken hepatic inflammation. The mechanisms responsible for this amelioration are multiple: increased Muc2 and occluding expression in the gut barrier, indicating recovery of the barrier integrity, and decreased LPS serum levels and TLR4 expression in the liver. The authors suggest that this amelioration due to *Roseburia spp*. is mediated by TLR5 (128).

Animal models of alcohol-induced liver injury indicate that TLR2/Myd88 and TLR9/Myd88 signaling in hepatocytes is indispensable for neutrophil infiltration and liver injury. Following depletion of KCs, infiltration of neutrophils is not prevented, however ALT levels and the gene expression of proinflammatory cytokines, such as *Il1b, Il6*, and *Tnfa* is decreased in KC-depleted mice compared with controls. This study suggests that dysbiosis in alcohol-related liver disease could increase hepatic presence of TLR2 and TLR9 ligands, thus contributing to liver inflammation (129). The activation of TLR2 pathway in KCs seems to be equally deleterious in the progression of alcohol-related liver disease and its deletion was beneficial for mice, due to a decreased activation of the NF-κB pathway and increased release of the anti-inflammatory cytokine IL-10 (130).

Activation of KCs via LPS-TLR4 pathways seems to be essential in the pathogenesis of alcohol-related liver diseases by activating a TRIF-dependent pathway and upregulating TNF-α (131). Bala et al. partly explain this by the increased activation of miR-155 in KCs following alcohol diet in mice. When induced, miR-155 negatively regulates inhibitors of the LPS-TLR4 pathway, thus enhancing KCs response to LPS (132).

#### NAFLD and NASH

Several studies in human or animal models have shown an association between changes in the gut microbiota and activation of TLRs signaling pathways in the liver (133, 134). TLR2, 4, 5, and 9 have been particularly associated with the progression of NAFLD and NASH, studies are summarized in Table 2.

**Table 2:** TLRs involvement in the progression of NAFLD and NASH.

	**NAFLD**	**NASH**
TLR2	↗ in HFD mouse KCs (135)	CDAA, expression by KCs increases inflammation and fibrosis, TLR2 activation is followed by inflammasome activation IL1α and IL1β (136)
TLR4	↗ in HFD mouse KCs (135) ↗ in patients biopsies (135)	↗ in patients biopsies ↗ in patients serum (137) ↗ liver expression after DSS treatment in a NASH model (138) ↗ liver expression in a NASH model (108)
TLR5	Activated in hepatocytes and adipocytes in HFD model (139) ↗ in NAFLD patients	Expression in hepatocytes has a protecting role in NASH model (140, 141)
TLR9		↗ in patients biopsies (142) ↗ liver expression after DSS treatment (138) Expression by KCs neutrophils and DCs is involved in NASH progression (143) Deletion has a protective effect in a HFD model (143)

*CDAA, Choline deficient amino acid; DCs, Dendritic cells; DSS, Dextran sodium sulfate; HFD, High fat-diet; IL, Interleukin; KCs, Kupffer cells; NAFLD, Non-alcoholic fatty liver disease; NASH, Non-alcoholic steatohepatitis; TLRs, Toll like receptors; ↗, Overexpression*.

Mridha et al. observed increased TLR4 and TLR9 mRNA in NASH patient biopsies compared to steatotic or control livers. Those results were confirmed in two murine NASH models, leading the authors to use *Tlr9*^−/−^ mice. They showed that these mouse liver exhibit steatosis in the same way WT mouse do under HFD, however they seem to be more protected toward liver inflammation and fibrosis. Characterization of the inflammation revealed that liver from *Tlr9*^−/−^ mice have a diminution in NF-κB and JNK activation and less macrophage and neutrophil recruitment compared to WT when fed with a high sucrose (atherogenic) diet. Moreover, evidence indicate that bone marrow derived cells expressing TLR9 are responsible for macrophage and neutrophil recruitment in a NASH model and that *Tlr9* deletion allows a reduction of hepatic inflammation (142).

TLR6 implication in the pathogenesis of liver diseases is mostly unexplored. In a NAFLD cohort, Arias-Loste et al. observed an overexpression of TLR6 in monocytes isolated from NAFLD patients compared to controls and a similar overexpression in monocytes, T cells and B cells of NASH patients compared to NAFLD. Following *in vitro* stimulation with TLR2 and TLR6 agonists, those isolated monocytes produce more pro-inflammatory cytokines like IL-1β, TNF-α, and IL-6. Furthermore, TLR6 expression is also increased in the liver biopsies of NAFLD and NASH patients. These results suggest a role of TLR6 in the progression of NAFLD to NASH and its potential use as a new marker in patient blood samples (144).

HFD in a mouse model is responsible for an increase of flagellin producing bacteria species in the gut, which causes an increase in serum LPS and hepatic flagellin presence. Flagellin in the liver activates TLR5/Myd88/NF-κB pathway in hepatocytes, then responsible for elevated HDL cholesterol levels (145). Moreover, activation or TLR5 in hepatocytes by flagellin increases intrahepatic CD8^+^ T cell response, possibly through secretion of IFN-γ (146). Munakka et al. previously demonstrated that flagellin induced TLR5 in adipocytes was responsible for accumulation of fat in hepatocytes of mouse fed with HFD. Moreover, they showed that adipose tissue TLR5 expression correlated closely with liver fat content in NAFLD patients (139).

#### Cirrhosis

TLR2 is upregulated in PBMCs of cirrhotic patients displaying high serum LPS levels (147). In order to mimic cirrhotic patient's tendency to develop bacterial infections, Hackstein et al. used a combination of BDL and carbon tetrachloride (CCl_4_) as a cirrhosis model to observe the anti-microbial capacity of immune cells when they induced bacterial infections. Under these conditions, they demonstrated that immune cells, in particular liver-resident macrophages, are unable to control bacterial infection compared to control mice. The authors observed a decreased expression of IL-1β, IL-12, and IFN-γ, known for their anti-microbial properties and increased production of TNF. Moreover, following bacterial translocation to the liver, IFN-β expression is enhanced in the livers of cirrhotic patients vs. healthy controls and in macrophages and DCs of BDL mice compared to sham. *In vitro*, this overexpression occurs following activation or TLR2, 4, and 9 pathways, which will trigger IL-10 secretion by isolated macrophages (148).

#### HCC

In an HCC mouse model, TLR4 deficiency slows down the progression of hepatic tumors and decrease F4/80^+^ immune cell infiltration. Those immune cells were characterized as hepatic macrophages, and when stimulated with LPS through TLR4 signaling, they will produce pro-inflammatory cytokines such as IL-6 and TNF-α, thus contributing to tumors growth. Hepatic macrophages involvement in the progression of HCC in this mouse model was further demonstrated by the fact that depletion of those macrophages suppressed tumor growth (149). The downregulation of miR-143 in HCC tumors from patients is responsible for an increased proliferation of cancer cells associated with less apoptosis. Authors suggest that this loss miR-143 is causing the overexpression of TLR2 in tumors and thus the activation of the NF-κB pathway (150).

Mularczyck et al. recently showed the role of Fetuin-A (Fet-A), an α2-Heremans-Schmid glycoprotein (AHSG), in the activation of the TLR4-JNK-NF-κB pathway in human hepatocarcinoma cell line (151). Once activated, this TLR4 pathway will aggravate insulin resistance and participate to NAFLD progression. In this study, the established a protective effect of probiotics emulsion against lipotoxicity and apoptosis in HepG2 cell lines. Authors showed that this protective effect is mediated through the regulation of Fetuin-A-TLR4-JNK-NF-κB axis thus preventing an increase of insulin resistance.

### Other Non-TLRs Members of the PRR Family

Dectin-1/Clec7a is a C-type lectin receptor member of the PRR family and recognizes β-glucans from pathogenic fungi. Dectin-1 is expressed by myeloid cells including NK cells, macrophages, DCs and neutrophils, where it can induce the secretion of several pro-inflammatory cytokines and chemokines and modulate inflammation *in vivo* (152). Dectin-1 is overexpressed in human and mouse liver fibrosis. In a CCl_4_ mouse model, the upregulation of Dectin-1 in hepatic DCs and macrophages is responsible for the downregulation of TLR4 and CD14, which negatively regulates liver fibrosis, inflammation, and HCC development (153). Indeed, following deletion of Dectin-1 mice are more prone to develop liver fibrosis and tumors. The authors observed a massive infiltration of leukocytes, neutrophils and bone marrow derived macrophages compared to control group. Furthermore, higher levels of IL-6 and TNF-α are detected in the KO mice (153). Yang et al. described fungal overgrowth and increased plasma levels of β-glucan in mice fed with ethanol. The use of antifungal treatments decreases liver injury and steatosis following ethanol feeding. The authors demonstrated that elevated β-glucan levels are responsible for the Dectin-1 dependent secretion of IL-1β by Kupffer cells and activation of NRLP3 pathway (26). Increased presence of *Candida* species in PSC patients bile suggest a role for Dectin-1 in the progression of the disease (154).

Galectin 3 is a member of the galectin family who recognize a variety of microbial pathogens (viruses, bacteria, fungi) and is expressed by neutrophils, macrophages, DCs. Galectin 3 specifically binds to β-1,2-mannans from *C. albicans* and can induce its death alone *in vitro* (155) but galectin-3 can also be secreted by neutrophils, link to pathogens and induce their phagocytosis by neutrophils. Primary macrophages expressing TLR4 and CD14 secrete galectin-3, which binds to LPS and negatively regulates LPS-driven inflammatory cytokines (IL-12, IL-6, and TNF-α) release (156). Besides, Jouault et al. showed that co-expression of TLR2 and galectin-3 is required for the endocytosis of *C. albicans* and the release on TNF-α by macrophages (157).

Pro-inflammatory IL-1β can be produced following TLRs pathway activation but also after the NOD-like receptor NLRP3 activation. Inflammasome expression is found in innate immune cells including monocytes, macrophages, neutrophils, and dendritic cells, NLRP3 inflammasome remains the most studied and has been involved in the progression of several chronic liver diseases (158). IL-1β secreted by KCs in alcohol-related liver disease mouse models aggravates steatosis and fibrosis. However, in a similar alcohol-related liver diseases mouse model with a deficiency for NLRP3, steatosis, inflammation, IL-1β expression, and number of activated natural killer T cells are all decreased, highlighting NLRP3 role in the pathogenesis of alcohol-related liver disease. Activation of both NLRP3 and NLRP6 contribute to dysbiosis, liver inflammation, and fibrosis via its effector IL-18 has also been demonstrated in several NASH studies. However, whether they display a protective or aggravating role remains unclear (159–162). NLRP3 expression is enhanced in NASH patients' liver, and seems to play a pro-inflammatory role in the progression from NAFLD to NASH in an animal model (163).

NOD1 and NOD2, two cytoplasmic receptors activated by fungi and bacterial peptidoglycan, are involved in the progression of liver inflammation by activating NF-κB and pro-inflammatory cytokines secretion in animal models, but their role in human liver diseases is mostly unexplored (164).

### Serotonin

The involvement of serotonin and the nervous system has been highlighted in a recent study conducted by Ko and co-authors. Indeed, by using a neural blockade approach they demonstrated that neural signal transduction from the liver via the visceral nerve is responsible for liver and body weight increase in HFD-fed mice and liver weight gain in CDAA-fed mice (165). They showed that nerve blockade could have anti-steatotic and anti-fibrotic effects in 4 week CDAA- and HFD NASH models. The authors suggest that these effects are mediated byan increase of Claudin-1 expression in intestine, as well through nerve blockade that potentially influences microbiota diversity and composition of SCFAs possibly slowing down progression of NAFLD. Moreover, they provide direct evidence that the nerve blockade decreases the expression of serotonin, a gastrointestinal hormone known for its regulating role in hepatic regeneration, thus suggesting that the influence of the visceral nerve on NAFLD progression in HFD and CDAA-fed mice models is partially a result of serotonin effect (165).

## Therapeutic Interventions Targeting the Gut-Liver-Axis

As stated above, the consistently growing understanding of the crosstalk between the gut microbiota and the liver offers multiple opportunities for novel treatment strategies (6). Currently, many therapeutic strategies to restore gut-liver axis homeostasis, rely on the modulation of the gut microbiome. Traditionally, this has been mainly achieved by using antibiotics, prebiotics, probiotics and fecal microbiota transfer (FMT), but intensive research over the last years has also resulted in a number of new approaches, including but not limited to managing alterations of bile composition.

### Application of Antibiotics

Treatment with antibiotics is the most common approach to modulate the intestinal microbiota and decrease bacterial infections. Due to their bactericidal action, non-absorbable antibiotics that mainly remain within the gut are often used to reduce the number of bacteria (and their metabolites). Especially in the context of small bacterial overgrowth, treatment with antibiotics provides an effective solution. Rifaximin is a broad-spectrum antibiotic and is currently widely used in patients with liver cirrhosis to prevent hepatic encephalopathy (166). In addition, it has been suggested that Rifaximin might also have beneficial effects on the gut microbiome and reduces intestinal permeability thereby protecting from the progression of cirrhosis via the gut-liver axis (166). Fujinaga et al. could recently show that the combination of Rifaximin with Angiotensin-II receptor blockers suppressed hepatic fibrosis in a mouse NASH model (167). In addition, a combined use of different antibiotics (e.g., Polymyxin B and Neomycin) was shown to prevent fructose-induced hepatic lipid accumulation in mice (168). Some antibiotics may also induce eubiotic changes and promote the growth of beneficial bacteria (e.g., *Bifidobacteria* and *Lactobacilli*) making them an attractive therapeutic option (169).

Promising preclinical data has resulted in numerous clinical trials focusing on the use of antibiotics for the treatment of liver diseases (see Table 3).

**Table 3:** Selected clinical trials targeting the gut-liver-axis for the treatment of liver diseases.

**Intervention**	**Study description**	**Results**	**Identification**
Antibiotics	Administration of Rifaximin in NAFLD patients	Reduction of serum endotoxin levels and improvement of insulin resistance, proinflammatory cytokines, and NAFLD-liver fat score (170)	NCT02884037
	Rifaximin + different doses of Simvastatin in patients with decompensated cirrhosis (Phase II)	No significant differences. 20 mg/day Simvastatin plus Rifaximin is recommended (171)	NCT03150459
FMT	FMT for the treatment of PSC (Phase I/II)	Increased diversity of the microbiome	NCT02424175
	Effect of FMT on NAFLD (Phase IV)	No results yet	NCT04465032
	FMT in cirrhosis (Phase I)	Reduced systemic inflammation and improvement of cirrhosis (172)	NCT03152188
	FMT for alcohol misuse in cirrhosis (Phase I)	FMT is safe and was associated with reduction in alcohol craving and favorable microbial changes	NCT03416751
Prebiotics	Supplementation with oligofructose in NASH patients	Improvement of liver steatosis (173)	NCT03184376
Probiotics	Multi-strain probiotic in NAFLD patients	Reduction of liver fat, serum AST and cytokine levels (174)	NCT03434860
	Effects of the Administration of *A. Muciniphila* on Parameters of Metabolic Syndrome	*A. muciniphila* improved insulin sensitivity and plasma total cholesterol (175)	NCT02637115
Synbiotics	Fructo-oligosaccharide + 4 bacteria in NASH (Phase II/III)	Reduction in hepatic steatosis and fibrosis	NCT02530138
	Supplementation with *Bifidobacterium animalis* and inulin in NAFLD patients	Improved liver enzyme concentrations and hepatic steatosis (176)	NCT02568605
Bile acid regulation	REGENERATE, OCA in NASH patients (Phase III)	OCA significantly improved fibrosis and key components of NASH (177)	NCT02548351
	Aramchol (fatty acid-bile acid conjugate) in NAFLD patients (Phase II)	Aramchol is safe, tolerable, and significantly reduces liver fat content in patients with NAFLD (178)	NCT01094158
	norUDCA in the treatment of PSC (Phase II)	Reduction of alkaline phosphatase levels	NCT01755507
	norUDCA in the treatment of NASH	Reduction of serum ALT (179)	not available
Duodenal mucosal resurfacing	DMR in the treatment of Type 2 Diabetes/NAFLD	Improvement of glycaemic control and reduction of liver fat content (180)	NCT02879383
	DMR in the Treatment of NASH patients	No results yet	NCT03536650
Carbon nanoparticles	Yaq-001 in patients with NAFLD	No results yet	NCT03962608
	Yaq-001 in patients with cirrhosis	No results yet	NCT03202498
Engineered probiotic	SYNB1020 in hepatic insufficiency and cirrhosis patients (Phase I/II)	Terminated due to a lack of efficacy	NCT03447730
Postbiotics	Supplementation with SCFA and physical activity in liver cirrhosis	No results yet	NCT03892070

*ALT, Alanin-aminotransferase; AST, Aspartate-aminotransferase; DMR, Duodenal mucosal resurfacing; FMT, Fecal microbiota transfer; NAFLD, Non-alcoholic fatty liver disease; NASH, Non-alcoholic steatohepatitis; norUDCA, 24-norursodeoxycholic acid; PSC, Primary sclerosing cholangitis; SCFA, Short chain fatty acids; OCA, obeticholic acid*.

### Fecal Microbiota Transfer

Fecal microbiota transfer (or transplantation, FMT) is a method to restore homeostasis of the gut microbiome by recolonizing the intestine with fecal bacteria from a healthy individual that has been studied extensively in infections with *Clostridioides difficile*. Several animal studies have also suggested beneficial effects of FMT in the progression of liver diseases (181). FMT could further prevent intestinal dysbiosis and alcohol- induced liver injury in mice and additional analysis showed an association with several species as *Bacteroidales, Clostridiales*, and *Enterobacterialis* (182). However, due to a mixed and individual composition of fecal microbiota, heterogeneous results have been reported so far. Nonetheless, currently multiple clinical trials focus on the use of FMT for the treatment of liver disease (see Table 3). In patients with metabolic syndrome, FMT from metabolic syndrome donors temporarily decreased insulin sensitivity, whereas after FMT from healthy donors, insulin sensitivity was not altered. Those findings were accompanied by alterations in intestinal transit time, inflammatory markers, fecal bile acid composition as well as changes in several intestinal microbiota taxa (183). Also in PSC patients, a recent clinical trial could demonstrate an improvement of microbial diversity in line with a reduction of alkaline phosphatase levels after FMT from a healthy donor (184). In a small but randomized controlled pilot study, Bajaj et al. further found beneficial effects of FMT on patients with cirrhosis and alcohol use disorder. FMT significantly reduced alcohol craving, and a reduction of serum IL-6 and LPS-binding protein could be observed (185).

### Prebiotics, Probiotics, Synbiotics

As opposed to antibiotics and FMT that aim to reshape the entire gut microbiome, specific changes in the microbiota can also be achieved through pro- and prebiotics or a combination of both (synbiotics). Probiotics are living microorganisms contained in food or supplements that may provide numerous health benefits (e.g., by promoting anti-inflammatory effects of the gut microbiota). In mice, the administration of probiotics (namely *Bifidobacteria, Lactobacilli*, and *Streptococcus thermophilus*) could reduce HFD-induced hepatic steatosis and insulin resistance and resulted in increasing hepatic NKT cells (186). Interesting *in vitro* studies with human hepatocarcinoma cells further suggested protective effects of probiotics against inflammation and obesity via reduction of Fetuin-A/TLR4-JNK-NF-κB pathway activation (151). Probiotic mixtures were also reported to reduce HCC growth in mice by modulating gut microbiota and resulting in a decrease of Th17 cells in the tumor (187). In a model of alcoholic steatohepatitis, it was shown that colonization with *Akkermansia muciniphila* was able to enhance expression of tight junctions in intestinal epithelial cells, thus decreasing intestinal permeability and systemic LPS levels (188). Those findings are in line with a recent clinical study, in which the supplementation with *A. muciniphila* led to an improvement in blood lipids and insulin sensitivity in obese patients (175). Supplementation with probiotic bacteria was also shown to improve the response to immunotherapy in cancer patients (189). However, the precise mechanisms involved in the beneficial effects of probiotics on HCC patients need to be elucidated.

Prebiotics are indigestible or low digestible fibers that can improve gut peristaltic and promote growth of beneficial bacteria. Prebiotics for example include oligosaccharides, polyunsaturated fatty acids or polyphenols. In mice, the treatment with pectin was shown to introduce major modifications of the intestinal microbiota and prevented steatosis and liver inflammation in alcohol-induced liver injury (182). Moreover, the administration of Inulin was found to prevent NAFLD via modulation of the gut microbiota (e.g., increase of *Akkermansia* and *Bifidobacterium*) and reduction of hepatic macrophages (190).

Due to the many beneficial effects of pre- and probiotics, novel studies suggest their combined use. Hadi et al. suggested that the consumption of synbiotics may improve plasma lipid concentrations and thus may have beneficial effects in the treatment of metabolic liver disease (191). As the treatment with pre- and probiotics is simple and has limited side effects, many clinical studies focus on their use in therapy for different liver diseases (Table 3).

### Bile Acid Related Therapies

Due to the diverse effects of bile acids on metabolism and the immune system, modulation of bile acid signaling is an attractive therapeutic option. Several FXR and TGR5 agonists as well as synthetic bile acids are currently under investigation to treat liver diseases. For example, obeticholic acid, a potent FXR agonist is currently used to treat PBC and has also shown many beneficial effects in the treatment of NAFLD/NASH and PSC (192, 193). In cirrhotic mice, treatment with FXR agonists appeared to improve intestinal barrier function by an increased expression of tight junction proteins and an increase of goblet cells (194). Currently, various agonists of the FXR receptor are in clinical phase II and phase III trials to test their efficiency in different liver diseases (Table 3).

Especially in PSC and PBC patients, the use of synthetic bile acids, as ursodeoxycholic acid (UDCA) is a well-established treatment strategy (195). Treatment with UDCA has also been shown to resolve intestinal dysbiosis in PBC patients (17). Moreover, norUDCA, a new homolog of UDCA, has shown promising results in the treatment of PSC as well as NAFLD patients (179, 195).

### Alternative Approaches Under Development

Novel upcoming therapeutic approaches to target the gut-liver-axis have recently been reviewed by Albillos et al. (6). Direct PRR targeting also holds promises for the development of future therapeutic options. Preclinical evidence suggests that small molecule inhibitors for TLR4 ameliorate liver failure in rodent models (196). In mice, Eritoran, a TLR4 antagonist, was further shown to attenuate hepatic inflammation and fibrosis (197). However, the relevance and potential side-effects of PRR targeting for liver disease treatment must be further investigated. Another promising approach consists of using adsorbant carbon nanoparticles that can ameliorate liver injury through absorption of gut-derived toxins and other bacterial products. In rodents with NAFLD, administration of a novel carbon nanoparticle, Yaq-001, resulted in a significant reduction of serum ALT and hepatic TLR4 expression (198). Yaq-001 is currently evaluated for safety and tolerability in patients with NAFLD and cirrhosis (NCT03962608 and NCT03202498).

Further preclinical investigations suggest that bacteriophages targeting specific bacteria may serve as a method to modulate the intestinal microbiota in a targeted manner. A recent study found that bacteriophages were able to specifically target intestinal bacteria and thereby attenuate alcohol-related liver disease (56). However, bacteriophage treatment is still in the explorative stage and further investigations are necessary, notably to evaluate its biosafety.

Metabolic diseases can further be treated by duodenal mucosal resurfacing (DMR), an endoscopic technique to ablate the duodenal mucosa and thereby improve glycemic and weight control (180). As studies have further reported an improvement of glucose homeostasis and transaminase levels, a possible improvement of NASH is also assumed. The effect of DMR to treat NASH is currently evaluated (NCT03536650).

Moreover, a lot of the recent research focuses on the identification of microbial metabolites that may have beneficial effects on intestinal barrier function (199). Postbiotics comprise all active compounds produced by intestinal bacteria, and include for example SCFAs, proteins, extracellular polysaccharides or organic acids. Recently, a novel postbiotic from *Lactobacillus rhamnosus* could show beneficial effects on intestinal barrier function and also potential protection of liver injury (200). Another recently published study evaluated the anti-inflammatory effects of *E. coli Nissle1917* derived metabolites and found TNF-α production as well as TLR signaling pathways to be reduced (201).

Further promising preclinical approaches to target the gut-liver axis include the application of engineered probiotics (e.g., SYNB1020) or synthetic live bacterial therapeutics (202, 203). Engineered and optimized bacteria were shown to be able to reduce ethanol-induced liver disease in mice (204).

## Conclusions and Future Perspectives

Cumulating evidence from the past years or decades indicate that the gut-liver axis is a promising therapeutic target to treat patients suffering from chronic liver diseases. Indeed, in a pathological context, gut-derived PAMPs and recirculating modified bile acids reach the liver, where hepatic and immune cells can recognize them through PRRs and thus exacerbate a pre-existing hepatic inflammation. Traditional approaches such as pre- and probiotics, FMT, antibiotics, and FXR agonists have already been or are being evaluated in numerous clinical trials. Further translational research will be necessary to transfer the new insights from innovative preclinical approaches to a clinical setting. Finally, approaches aiming at specifically altering targeted gut microbiota species, bile acids, cytokines and chemokines, will advance our understanding of the mechanisms involved in disease progression or resolution, and greatly advance therapeutic options.

## Author Contributions

AB and JH wrote the manuscript. AG designed the figures. AG and FT revised the manuscript. All authors approved the final manuscript.

## Funding

AB, JH, and FT were supported by the German Research Foundation, Grant Number DFG Project-ID 403224013, SFB 1382, and CRC/TR 296. AG is a recipient of a Humboldt Research Fellowship for Postdoctoral Researchers (Alexander von Humboldt Foundation).

## Conflict of Interest

FT's lab has received research funding by Allergan, Bristol-Myers Squibb, Galapagos, Gilead and Inventiva. He consults for Allergan, Bayer, Boehringer Ingelheim, Galapagos, Galmed, Intercept, Inventiva, NGM bio, Novartis, and Pfizer. The remaining authors declare that the research was conducted in the absence of any commercial or financial relationships that could be construed as a potential conflict of interest.

## Publisher's Note

All claims expressed in this article are solely those of the authors and do not necessarily represent those of their affiliated organizations, or those of the publisher, the editors and the reviewers. Any product that may be evaluated in this article, or claim that may be made by its manufacturer, is not guaranteed or endorsed by the publisher.

## Abbreviations

BDL: Bile duct ligation
CDAA: Choline-deficient L-amino-defined
DAMPs: Danger-associated molecular patterns
DCs: Dendritic cells
DSS: Dextran sodium sulfate
FMT: Fecal microbiota transfer
FXR: Farnesoid X Receptor
HCC: Hepatocellular carcinoma
HFD: High fat-diet
IBD: Inflammatory bowel disease
IFN: Interferon
IL: Interleukin
KCs: Kupffer cells
LPS: Lipopolysaccharide
NAFLD: Non-alcoholic fatty liver disease
NASH: Non-alcoholic steatohepatitis
PAMPs: Pathogen-associated molecular patterns
PBC: Primary biliary cholangitis
PRRs: Pattern-recognition receptors
PSC: Primary sclerosing cholangitis
SCFAs: Short-chain fatty acids
TLRs: Toll-like receptors
TNF: Tumor necrosis factor.
